# Parental singing during kangaroo care: parents' experiences of singing to their preterm infant in the NICU

**DOI:** 10.3389/fpsyg.2025.1440905

**Published:** 2025-02-04

**Authors:** Pernilla Hugoson, Friederike Barbara Haslbeck, Ulrika Ådén, Louise Eulau

**Affiliations:** ^1^Sachsska Children's and Youth Hospital/Södersjukhuset University Hospital, Stockholm, Sweden; ^2^Department of Neonatology, Newborn Research Zurich, University Hospital Zurich, Zurich, Switzerland; ^3^Department of Women's and Children's Health, Karolinska Institutet, Stockholm, Sweden; ^4^Department of Neonatal Medicine, Karolinska University Hospital, Stockholm, Sweden; ^5^Department of Bioclinical Sciences, Linköping University, Linköping, Sweden; ^6^Department of Nursing Science, Sophiahemmet University, Stockholm, Sweden; ^7^Department of Clinical Science and Education, Södersjukhuset, Karolinska Institute, Stockholm, Sweden

**Keywords:** emotional attachment, kangaroo care, music therapy, NICU, parental singing, patient and family centered care, preterm infant

## Abstract

**Introduction:**

Singing fosters emotional connections, attachment, bonding, and language development in infants. Prematurely born infants, however, are at risk of missing this vital communication, impacting neurodevelopment and family wellbeing, especially during prolonged hospital stays. Kangaroo care provides physiological and emotional support, while Creative Music Therapy (CMT) has demonstrated positive effects on neurodevelopment, parental wellbeing, and attachment. The Singing Kangaroo project, a Swedish-Finnish multi-center randomized controlled trial (RCT), investigated the impact of parental singing during kangaroo care. This qualitative follow-up study explores these findings through the lens of Antonovsky's Sense of Coherence (SOC) model.

**Method:**

Semi-structured interviews were conducted with 28 families (20 intervention group, eight control group) at their infant's 5-month corrected age. The intervention group received CMT twice weekly during kangaroo care for 4 weeks in the NICU, while the control group received standard care. Data were analyzed inductively, followed by deductive categorization within the SOC framework, focusing on its three core components: Manageability, Comprehensibility, and Meaningfulness.

**Results:**

Parents in the intervention group reported enhanced understanding of how singing fosters attachment and boosts their self-esteem, aligning with increased manageability and comprehensibility. Control group parents also experienced joy in singing, which positively influenced family wellbeing, albeit less extensively. Across both groups, singing was described as a meaningful activity that strengthened parent-infant bonding and promoted emotional connection within the family.

**Conclusion:**

Parental singing during kangaroo care, particularly when supported by a trained music therapist, enhances parents' sense of coherence by fostering comprehensibility, manageability, and meaningfulness. This study highlights the long-term benefits of integrating CMT into family-centered NICU care to support both infants' neurodevelopment and family wellbeing.

## 1 Introduction

Most of the time it's just joy, like, that I want to draw out laughter from him and sing when playing, but I also feel a lot of closeness. When I sing lullabies, I feel very strong feelings of closeness and love and tenderness. It becomes like a way to hold him in a different way, you like hold him with music. You embed yourself in the lullaby!

*Mother from the intervention group*

All over the world, parents sing to their infants in all known cultures. It is a way for infants and parents to connect emotionally when building relationships. Parents use their voices in different ways; they sing, hum, vocalize and use their voice in smooth and soft melodic small talk, so-called infant-directed speech, when communicating with their infants (Bonnár, 2014; Sharman et al., 2023). The infants interact in non-verbal communication via movements, gestures, facial expressions and sounds (Malloch and Trevarthen, 2018). *Communicative musicality*, as described by Malloch and Trevarthen (2009), refers to the delicate and intimate exchanges between infants and their parents. This form of communication is essential for parent-infant interaction, supporting attachment—the emotional connection that develops over time through consistent care—and bonding, the immediate emotional tie formed shortly after birth. Such interactions are fundamental for fostering secure attachment and strengthening the parent-infant bond (Beebe, 2017; Trevarthen, 1999). Furthermore, early reciprocal communication, enhanced by musical elements, plays a crucial role in the infant's socio-emotional development and lays the foundation for early language acquisition (Falk and Audibert, 2021; Jaffe et al., 2001).

Regarding premature-born infants, attachment, bonding, and language development can be suppressed. Preterm infants are at risk of neurodevelopmental (Pascal et al., 2018), language (Vandormael et al., 2019) and cognitive developmental impairments (Aarnoudse-Moens et al., 2009). The parents are confronted with psychosocial challenges during their often long-lasting hospital journey (Galea et al., 2022). These challenges can compromise their intuitive parenting skills and their ability to provide their fragile infant with the intimate closeness and safety that only they, as parents, can give (Flacking et al., 2012; Sanders and Hall, 2018). In addition, preterm infants' social behavior is still immature, and infant cues are much harder to understand than in full-term babies, which may suppress the natural parent-infant-interplay as well (Porges, 2011).

A way to address both infants' and parents' needs is to provide developmentally supportive child and family-centered care. This way of caring for the families in the neonatal intensive care unit (NICU) has become standard in many countries worldwide. It strives to put the family at the center of care and the infant at the center of the family (Bronfenbrenner, 1979; Darling, 2007; Kuo et al., 2012; Kutahyalioglu and Scafide, 2023). An essential part of this is kangaroo care, which supports the infant's physiological needs but also the emotional needs of both the baby and the parents (Clarke-Sather et al., 2023; Narciso et al., 2022; Pathak et al., 2023). It gives the infant direct access to the parent's bare chest, heartbeat, breathing, smell, warmth, vibrations from the bone construction, and the parents' voice. It has been shown to stabilize the preterm-born infant's respiratory rate, oxygenation and temperature and to support breastfeeding (Boundy et al., 2016). Furthermore, kangaroo care is associated with reduced maternal depression and anxiety and improved parental wellbeing, bonding and attachment (Pathak et al., 2023).

Singing and speaking during kangaroo care seem to be one of the most natural ways to connect and communicate between infant and parents (Filippa et al., 2017; Hugoson and Eulau, 2023). Indeed, parents sing and use their voices when interacting with their preterm infant in the NICU if they feel it safe to do so (Shoemark and Arnup, 2014). Parental singing during kangaroo care may improve the infant's autonomic stability and decrease maternal anxiety (Arnon et al., 2014; Kostilainen et al., 2020). Further, it has been associated with enhanced auditory development, an integral part of early language development (Kostilainen et al., 2021; Partanen et al., 2022). Parents experiences of singing to their preterm infant in the NICU have been shown to have a positive impact on shaping parental identity, meeting parents' emotional needs and acting as a way for parents to connect with their infant (Ettenberger et al., 2016; Ghetti et al., 2021; McLean, 2016; McLean et al., 2018; Palazzi et al., 2020). Notably, the music therapy method Creative Music Therapy (CMT) is associated with improved neurodevelopment in the infant (Haslbeck et al., 2020), parental wellbeing and fostered attachment and bonding between the preterm infant and the parents (Haslbeck, 2014; Haslbeck et al., 2021; Kehl et al., 2021).

Creative music therapy with preterm-born infants and their parents (CMT) is a family-centered resource and needs-oriented, well-established and researched music therapy method (Haslbeck and Bassler, 2020). A certified music therapist gently supports the infants' need for a sensitive, sensory, meaningful experience and the parents' need to communicate interactively with their preterm infant. The aim is to strengthen the intuitive capacities of parenting by supporting and empowering the parents in an undemanding and relational therapeutic approach while sharing the experience of the infants' expressions here and now guides the dialogue between parents and the music therapist.

### 1.1 Singing kangaroo study

In an ongoing multi-center study, the Singing Kangaroo, Finnish and Swedish researchers explored the potential benefits of parental singing on the language and neurodevelopment of prematurely born infants, as well as the impact of parental singing on the wellbeing of the families (ClinicalTrials.gov ID NCT03795454). In the Finnish cohort, positive results of parental singing have been found regarding maternal wellbeing, early mother-infant relationship and early auditory discrimination of speech sounds in the infants (Kostilainen et al., 2020, 2021).

The Swedish cohort began by conducting a randomized controlled trial (RCT) assessing the impact of parental singing during kangaroo care on auditory processing of standardized audio stimuli using magnetoencephalography (MEG) at term-corresponding age (Partanen et al., 2022). Preterm infants (born between 24 and 32 weeks of gestation) admitted to the NICU were randomly assigned to the singing intervention group or the control group. The intervention group, implementing CMT, received gentle support from a qualified music therapist twice a week for 4 weeks, to sing or hum in an infant-directed manner during kangaroo care. The control group received standard kangaroo care. Both groups documented their singing frequency and duration throughout the day and night in diaries, collected weekly by the music therapist, fostering ongoing communication. The design of the diaries was based on the one used by Raiskila et al. (2017), with an added parameter for parental singing.

The MEG findings indicate that infants in the singing intervention group exhibit larger neural responses to changes in speech sounds compared to those in the control group, even when accounting for the overall amount of singing during kangaroo care in both groups (Partanen et al., 2022). These results, in conjunction with the potential impact of CMT on positive parenting, meaningful interactions, empowerment, improved parental self-esteem, and enhanced caregiver sensitivity studied by Haslbeck (2014) and Haslbeck et al. (2021), raise questions concerning potential positive outcomes on the whole family when incorporating parental singing, gently supported by a qualified music therapist, into kangaroo care.

In light of the potential of CMT to mobilize parental resources, facilitate meaningful interactions, and promote parental empowerment, the concept of coherence emerged during the initial discussions between the first and last authors. When meeting major life events such as preterm birth, it is essential to find ways to manage the situation. Antonovsky (1979) presented the concept of *sense of coherence* (SOC) as a way of understanding how people undergoing stressful and potentially traumatic experiences can mobilize their resources. Sense of coherence consists of three key components: comprehensibility, manageability, and meaningfulness. Comprehensibility is the cognitive component that refers to how the individual takes in and understands the information given. Manageability is the component that refers to how the individual perceives whether their resources available are sufficient or not. Meaningfulness refers to the motivational component i.e., the extent to which the individual feels what they experience is worth it. These three components interact and are all needed to create a sense of coherence (Moksnes, 2021).

With a focus on parents' experiences and aiming for a deeper understanding of the mechanisms involved in the observed outcomes in Partanen et al. (2022), we proposed a qualitative follow-up study to further explore and unpack the benefits of parental singing in relation to the concept of coherence. Therefore, the aim of this study, as part of the Swedish cohort of the Singing Kangaroo project, was to explore the potential influence of CMT in the NICU on enhancing the sense of coherence in parents as they engage in singing to their preterm-born infants during kangaroo care in the NICU. The research questions arose about how the parents experience singing for their preterm-born infant during kangaroo care and whether CMT may influence the parental sense of coherence?

## 2 Method

This study adopts a qualitative interpretative design, utilizing a phenomenological hermeneutical method (Lindseth and Norberg, 2004) inspired by Ricoeur (1976), and grounded in a hermeneutic philosophical framework (Patton, 2015). The method integrates the life-world philosophy of phenomenology with the hermeneutic tradition of text interpretation to illuminate the meanings of lived experiences. These experiences, while inherently individual, were interpreted from interviews transcribed into autonomous text, enabling an understanding and explanation of human phenomena (Ricoeur, 1976; Lindseth and Norberg, 2004).

**Figure 1:**
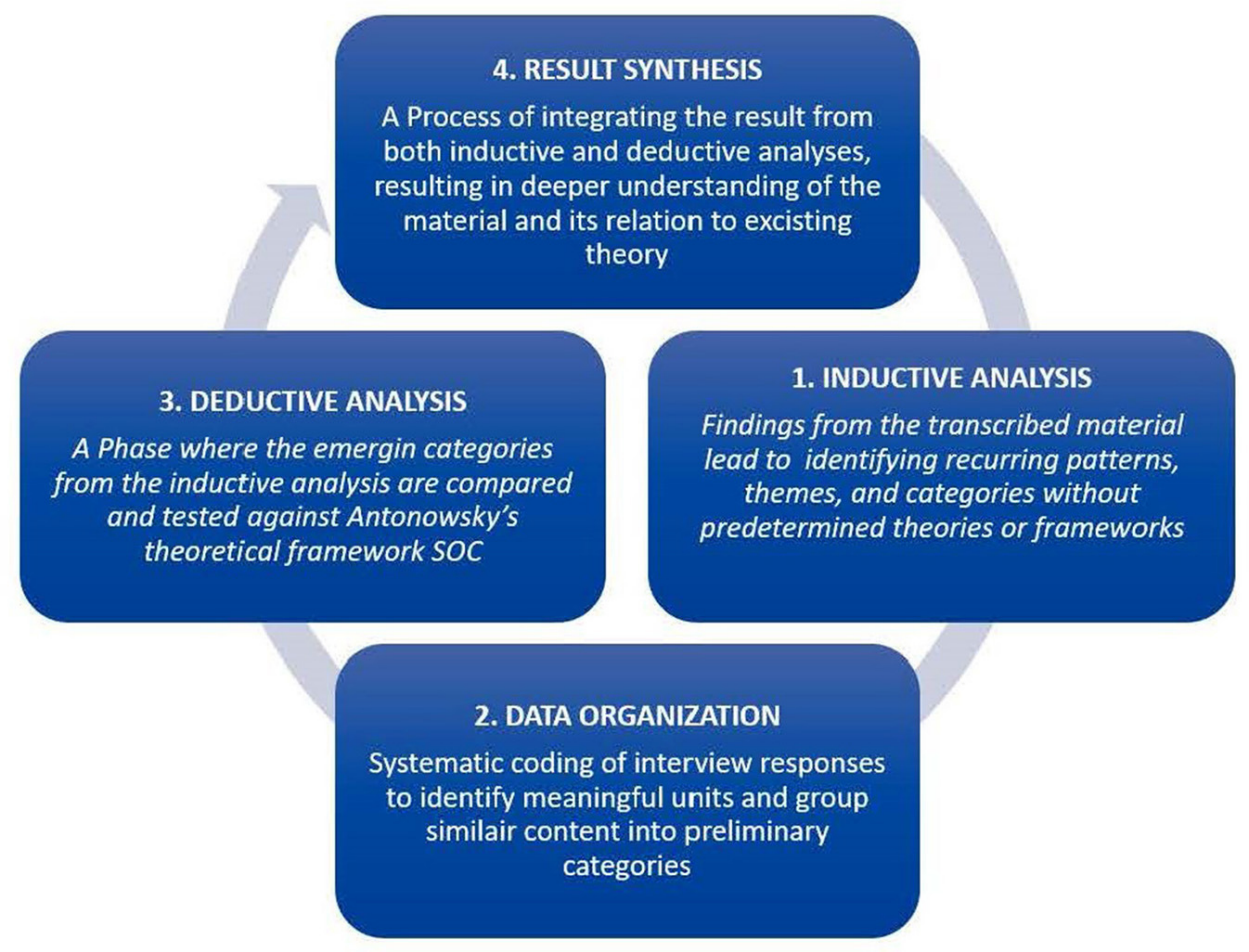
Outline of the data analysis process: beginning with inductive analysis to identify themes, transitioning to deductive analysis to test findings against a theoretical framework (e.g., SOC), and concluding with the synthesis of results.

The analysis was conducted using a manifest inductive qualitative content analysis (Elo and Kyngäs, 2008), emphasizing the interpretative nature of understanding human experiences. Semi-structured interviews were conducted with parents from two groups: an intervention group, who participated in the CMT (Creative Music Therapy) program, and a control group, who received standard care (Kvale and Brinkmann, 2009). The methodology combined inductive reasoning—synthesizing specific empirical observations into broader patterns and meanings—and deductive reasoning, where these patterns were examined against established theoretical frameworks (Flanagan et al., 2024). The hermeneutic perspective informed the dynamic interaction between these two processes, ensuring that interpretations were both grounded in the data and connected to existing knowledge (Figure 1).

### 2.1 Setting and participants

Families were recruited from two major hospitals in Region Stockholm (Hospital 1 and Hospital 2). Recruitment took place in conjunction with the RCT in which a total of 38 families were recruited (Partanen et al., 2022, see Section 1). Inclusion criteria were as follows: preterm infants born at Gestational Week (GW) 32 or earlier, clinically stable infants with parents fluent in either Swedish or English. Infants could be recruited at the earliest when the infant was 28 GW. After some lapses, 28 families remained available for interviewing. Of these, *n* = 20 were from the intervention group and *n* = 8 from the control group. As part of the recruitment, all families, in both groups, filled in a form concerning duration of skin-to-skin contact and singing (Kostilainen et al., 2023; Partanen et al., 2022). Among the interviewed families, 27 mothers (M) and 18 fathers (F) participated in the study. There were no same-sex relationships among the families. There was one single mother, and one single father. The interviews took place when the infant reached the age of 5 months corrected age. The decision to interview the parents several months after leaving the NICU/hospital and completing the intervention was made to assess whether the CMT intervention had any enduring effect on parents singing to their infant at home after discharge. This has also been studied in the interdisciplinary “Transition to home” study in Switzerland (Haemmerli et al., 2021; Schütz Hämmerli et al., 2022) and in an international longitudinal multicenter study (Gaden et al., 2022). In a recent integrative review the authors suggest that music therapy could play an important role also after discharge (Clemencic-Jones et al., 2024). In our study, interviews were conducted with parents from both the intervention and control groups.

In the Swedish cohort of the Singing Kangaroo RCT study, families in the intervention group met a CMT-trained music therapist (author PH) twice a week during 4 weeks of hospital stay. Each music therapy session lasted ~45 min and was provided during kangaroo care. The music therapy sessions were conducted in an individualized and interactive way where parents were gently supported and inspired to use their voice in humming, singing, and vocalizing with their preterm infant. The music therapist had an undemanding and relational psychotherapeutic approach when reaching out to the families and aimed to guide the parents gently to find their own unique voice in an undemanding approach. The music therapy method used was in accordance with the CMT method (Haslbeck and Bassler, 2020). The parents' preferred way to sing to their infant was in an infant-directed lullaby style: a tender and warm voice timbre, a slow tempo, humming a repetitive melody without words in accordance with their infants breathing and movements. The music therapy sessions were tailored to both parents' and their infants' needs in the moment. They could consist of singing songs together, humming and vocalizing together, sometimes accompanied by the monochord instrument. These were moments of peace and quiet when listening to the music therapist's singing or humming improvising in a relaxed lullaby style or listening to the soft sound of the monochord. What was happening in the moment was thus emphasized and illuminated. This created space for dialogue addressing parents' needs to share feelings and thoughts (Haslbeck and Hugoson, 2017).

### 2.2 Data collection

The semi-structured interviews, conducted by author PH responsible for the CMT at NICU occurred between May 2015 and November 2018, were audio recorded using an Olympus digital voice recorder VN-713PC. To ensure a comfortable environment, the sessions were conducted in the family's home. Interviews, held separately with mothers, fathers, or both parents together depending on the situation, often included the presence of infants. In two cases, fathers responded to questions via email due to personal constraints. To explore the parents' experiences, an interview guide with open questions and possible follow-up inquiries was co-designed (LE + PH) (see Appendix 1). The interviews were conducted in Swedish and took between 20 and 45 min each. After each interview the first author made field notes of personal impressions to remember better the context of the whole interview situation when listening, transcribing and analyzing the interviews.

### 2.3 Analysis

Interviews with parents (*n* = 17) from Hospital 1 were transcribed by the first author (PH) in an ongoing process during the data collection period. Transcriptions (*n* = 11) from Hospital 2 were made by a professional transcriber due to time limitations in January 2023. The interviews with the parents were transcribed verbatim, every pause and non-verbal utterance, such as laughs, sighs and humming were noted. The transcribed interviews were analyzed according to the method of Elo and Kyngäs (2008) using a manifest inductive qualitative content analysis. The data resulting from these interviews were then subjected to manifest qualitative content analysis using a deductive approach. The analysis began with several readings of the transcribed text to search for meaning and a deeper understanding of the entire data set. The first author (PH) and the third author (LE) read the transcripts separately because important insights can emerge from the different ways that people consider the same data (Sandelowski, 1998). The authors initially shared their impressions of the interviews and collaboratively identified meaning units aligned with the study's aim.

After reviewing the material from the transcriptions of the first hospital cohort, a decision was made to apply a deductive approach, explicitly using Antonovsky's Sense of Coherence (SOC) theory, for labeling the categories (Antonovsky, 1979). Meaning units were differentiated using colors and then transformed into condensed meaning units, codes, and finally, three subcategories and one main category, organized in an Excel document (see Tables 1, 2). PH initially did the categorization matrix translation (Tables 1, 2) and later refined it through a peer review process involving all authors. As two researchers (LE and PH) participated in the coding, and one of them (PH) held dual roles as a music therapist and researcher, they independently tested the coding before extensive discussions between the authors on the categories. Any disagreements were resolved through discussions until a consensus was reached. To enhance credibility and trustworthiness, the authors provided a comprehensive description of the content analysis process and presented results from the original data, enabling readers to draw their conclusions following the method proposed by Elo et al. (2014). Data analysis continued until a certain material saturation was reached when no new information was found in the material.

**Table 1:** Examples of content analysis, intervention group.

**Meaning unit**	**Condensed meaning unit**	**Code**	**Subcategory**	**Category**
I was able to calm myself down. If I was sad, which I feel I was quite often, or angry at someone, I thought that I felt better by singing. It felt…well, everything became more positive.	Singing made me feel better and I calmed myself down when things were difficult.	Comfort Wellbeing	Manageability	Sense of coherence
I feel that she becomes calm, that she enjoys hearing my voice. I think it's the response from her that makes me continue.	She becomes calm, she enjoys it. The response from my child provides motivation.	Wellbeing Communication Motivation	Comprehensi-bility	Sense of coherence
Then you came and you sang for us and with me and it went really well! […] I have been inspired by you and I sing much more for her since I met you. […] We also got a lot of emotional support!	It was supportive and inspirational to sing together with the music therapist and the emotional support was valuable.	Emotional support Inspiration	Meaningfulness	

**Table 2:** Examples of content analysis, control group.

**Meaning unit**	**Condensed meaning unit**	**Code**	**Subcategory**	**Category**
I didn't think it was so noticeable, but others said that they thought she reigned in and became calmer, but I don't know…it felt like I was doing it more for me!	The singing became something that I did to calm myself because I couldn't see that my baby reacted.	Relaxing	Manageability	Sense of coherence
The reason why you sing is perhaps to make the child feel safe and calm and so on and to recognize your voice.	I want my child to be safe and calm so I sing.	Reassurance	Comprehensi-bility	Sense of coherence
You get closer to each other when you do something together […] it made us think about something else, and I think we got closer to them […] it became a break from other things […] so it's also a good way to take a breath.	A closeness when singing together, a break and a way to breathe.	Togetherness Wellbeing	Meaning-fulness	Sense of coherence

### 2.4 Ethical considerations

The Swedish component of the Singing Kangaroo studies, encompassing the present study, obtained ethical approval from the Swedish Ethical Review Authority (registry number 2014/1318-31; supplementary approval for interviews, registry number 2015/0120-32, supplementary approval registry number 2016/2362-32). All participating parents provided written informed consent before participating in the study and prior to taking part in interviews. They were informed that participation was voluntary, and they had the right to withdraw at any time without providing a reason, and it would not impact future care for themselves or their infants. We randomized each family to a three-digit number code to ensure anonymization. Parents (mother = M, father = F) with the same number belong to the same family. The code list is kept in a looked cabinet to which only the research group has access.

### 2.5 Limitations and strengths

A limitation of this study is the potential for participation bias, as families with a pre-existing interest in music may have been more likely to participate, raising concerns about the representativeness of the clinical sample. Additionally, the unequal sizes of the intervention and control groups—a common challenge in qualitative research, as noted by Patton (2015)—may have affected the depth and breadth of the findings. Smaller control groups can impact the balance of data and require careful interpretation to ensure credible conclusions. Nonetheless, the dual-group approach enabled meaningful comparisons, highlighting both shared and unique parental experiences between the CMT and standard care groups. Another limitation was that the same researcher (author PH) conducted both the clinical intervention and subsequent interviews. While this dual role provided valuable contextual insights, it may also have influenced participants' responses. Patton (2015) underscores the importance of transparency in addressing such design decisions to maintain methodological rigor. To mitigate potential bias, both authors (PH and LE) independently analyzed the interview data during the initial phase, which strengthened the credibility of the findings.

This study employed a robust two-stage analytical approach, combining inductive analysis to identify patterns with deductive analysis grounded in the SOC framework (Antonovsky, 1979). As the first qualitative inquiry in music therapy within neonatal care, it provides valuable insights into parental experiences in both intervention and control groups. However, the significantly unequal group sizes limit generalizability, as variability in responses was constrained in the smaller control group, potentially influencing the findings. Future research should address these limitations to enhance comparability and rigor.

## 3 Result

**Figure 2:**
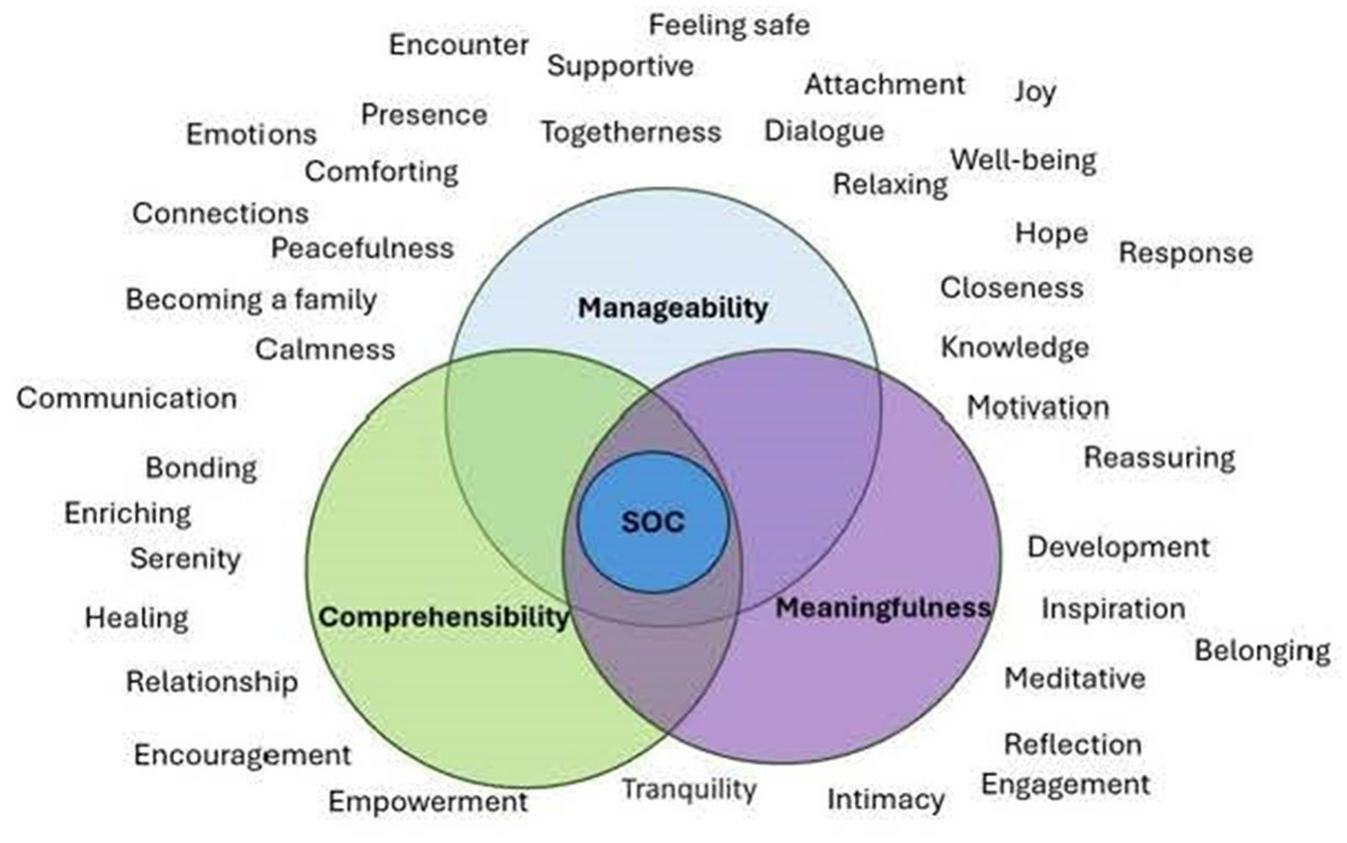
Venn diagram showing the possible interscetions between the three sets comprehensibility, manageability, and meaningfulness.

The results emerging from the inductive process revealed the themes of *feelings of safety, closeness, empowerment in parenting and healing*. The deductive analysis that followed, using the theory of SOC, resulted in three subcategories: *manageability, comprehensibility, meaningfulness—*the main categories of the concept *Sense of Coherence* (Figure 2). The subcategories coalesced into one main category: *Sense of Coherence* (see Tables 1, 2). A sense of coherence is achieved when manageability, comprehensibility, and meaningfulness are in harmony with each other. Each subcategory is illustrated with quotations to illuminate the categories with the parents' own words. To distinguish between the CMT-intervention group and the control group, the results are presented under separate subcategory headings.

The Venn diagram (Figure 2) illustrates the possible intersections between the three concepts of *Comprehension, Manageability, and Meaningfulness*. The overlapping regions between each circle visually depict shared similarities between the concepts. However, SOC occurs only within the region where all the circles intersect. The Venn diagram derives all the concepts “floating around” from the parents' interview responses.

### 3.1 Manageability

#### 3.1.1 Intervention group

The majority expressed difficulties in managing their situation in the NICU. Becoming a parent to a fragile and ill infant is not an easy task. They reported that singing with their infant helped them to be more present and find relaxation in those moments.

***348M:*** *I found it very comforting, quite liberating actually, to sink into, like, a song for a while, it was very relaxing! Both for me and for her [baby] It was incredibly stressful when we were in the emergency room… […] At that moment, it was good to be able to relax for a while*.

***551M:*** *But it's a way to communicate with him [baby], I'm calm and I want him to be calm while we're together here*.

Several mentioned feeling anxious about their infant and experiencing a sense of losing control, unable to care for their infant as they would like. For them, singing became a strategy for managing their situation.

***909M*** *: The song itself, for me, was initially a way to disconnect from all the chaos […]. It allowed me to focus on singing and being in the moment. Looking back, it was like small oases in a little chaos. Physically, I felt that I could relax in those moments when X. was upset and there was something going on, and then we sang!*

***236M:*** *I remember it as cozy to sing in the emergency room. It was like creating a private space […] Singing becomes like a mantra for yourself… I think I mostly hummed, actually…a way for him to hear my voice! Even if I didn't have the ability to talk to him, which might have been because everything was challenging*.

Additionally, some parents shared that singing helped them manage the stress experienced in the NICU, serving as a tool for comforting both themselves and, by extension, also their infant.

***551F:*** *Then I notice that it's [parental singing] a tool too, once you start using it… it's practical; you can calm him down, you can see that. Especially in the neonatal unit […] I found it easier to calm him down with a little song. […] you yourself [parent] become calm with the music, with your calmness, he becomes calmer too*.

A significant number of parents also noticed that singing was a way they could help their infants calm down during skin-to-skin contact, making them more content. This calming effect was evident, often causing infants to fall asleep instead of being restless.

***969*****+*983M:*** *They [babies] could be a bit restless when we had skin-to-skin contact – it was mainly at that moment we used to start singing. They often fell asleep instead of fidgeting around, especially the girl, I think, because she didn't really want to lie still. She was probably bothered by all the equipment and such, so I think it helped to calm her down*.

***551M**: I think it was a way for him [baby] to find tranquility amidst all the beeping and all the tubes and machines and everything… because he usually fell asleep… it was a cozy moment… which I believe he enjoyed too*.

Singing served as a meaningful and constructive activity for many parents, particularly those with infants enduring an extended hospitalization. It was described as a valuable way for parents to manage themselves positively during the challenging time at the NICU. Singing provided a helpful distraction, allowing several parents to focus on something other than the hospital environment and the challenges their infant was facing.

***969*****+*983F:*** *It was a way to occupy oneself! […] Especially for those who are hospitalized for a long time, it's very good that you get something else to do, you get a way that can help your children, it helps parents to think about something else*.

***132M:*** *It felt good to be able to do something for your child. […] we really felt that it was very important for us to sit a lot skin-to-skin and then that you could add one more thing, to sing and it was good!*

Certain parents mentioned struggling to feel fully present with their infant, as they also had to care for siblings at home. In this stressful situation, they found the music therapy sessions to be relaxing—a peaceful pause from all their duties.

***508M**: Yes, it was calming and distracting from all the other thoughts I had about organizing how we would handle things with my older son and how I would get to see him. Since you automatically smile when you sing, you feel more positive*.

***111F:*** *I think that in that situation you get really stressed. I don't think there is any other situation where you get so stressed: a child that was born prematurely and also like we had our second daughter. I think the best thing is to sing, it helps calm both you and the baby!*

Commencing singing in the NICU presented challenges for several parents, with feelings of embarrassment and initial discomfort commonly acknowledged. It also required a period of time for them to comprehend the benefits for their infant. Once this realization transpired, the task became more manageable and facilitated a stronger connection with their infant.

***930M**: It was a bit scary, and it probably has to do with the fact that I haven't sung much […] but it still felt good because it felt like it gave her something*.

***862F:*** *In the beginning, it was a bit tough, but then it got better […] it was a bit embarrassing at first, but it eased up after a while*.

#### 3.1.2 Control group

Most parents in the control group who sang to their infants found singing to help create a sense of manageability. Additionally, singing in the NICU became a playful and engaging coping mechanism in the challenging environment. Humming, particularly at night, served as a means to deal with hospital noise and fatigue.

***123*****+*724F:*** *It became the most playful in the NICU […] this singing activity was something different that we could engage in*.

***338M:*** *I hummed a lot at night in the hospital, there wasn't much you could do about this grunting and when there was a lot of noise, and noise like that, I stayed in bed and hummed because I was so tired, so I hummed instead*.

Certain parents in the control group contemplated that participating in a singing group, where they could gather inspiration and mutual support from other parents, would have constituted a favorable aspect during their hospital stay.

***338M:*** *[…] there could be a group where you could get positive input on what you can do and music could be part of that…and get the opportunity to meet the other parents also on a more positive basis*.

***776M:*** *You might be able to come to the parent lounge much like at an open preschool, so the parents and children who can join can be there and only hear music, that would be nice!*

One mother additionally expressed that it would have been beneficial if healthcare personnel had taken an active role in the singing process or provided guidance to parents on how to utilize singing as a coping mechanism in the NICU setting.

***776M:*** *Maybe the rest of the staff also have to encourage and support parents to come and that it works with the other care*.

To sum up manageability, the first key concept of SOC, the results from the intervention group indicate that parental singing served as a valuable strategy to calm the infant, particularly in the NICU. Parents highlighted its usefulness in soothing the baby and bringing a sense of calmness to themselves, especially in high-stress situations in the NICU. Singing was viewed as a practical and effective tool to manage stress, fostering a calming atmosphere for both them and the infant. In summary, singing served as a tool to manage tranquility and comfort, creating a positive and enjoyable experience for both infants and parents in the challenging NICU environment. It was perceived as a proactive way for parents to manage both their infant's wellbeing and their own wellbeing, adding a positive element to their hospital stay. Interestingly, only the parents of the control group expressed that they would have needed more support in singing.

### 3.2 Comprehensibility

#### 3.2.1 Intervention group

Comprehensibility is linked to knowledge and the capacity to contemplate emotions and thoughts, enabling parents to recognize their new parental role. As parents in the intervention group progressively became more acquainted with their infant and grew more at ease with singing, the situation became more comprehensible. This, in turn, facilitated a better understanding and acceptance of their situation.

***909M:*** *I think that before we met you [music therapist], it felt like… I probably didn't think of X as a child […] So for me, it was like, “Oh, you can also sing for such a small child.” Then he became a bit more human, and I've probably felt it even more during the autumn as he has grown, that now it feels even more natural to sing*.

***930F:*** *It felt good to do it [singing], it felt intimate, and like it maybe made one's presence more tangible for the child. I think it was good both for establishing a stronger connection and for getting the child accustomed to oneself while providing more comfort by marking one's presence*.

Music therapy sessions proved to be enlightening for some parents, providing insight into their infant's distinct communication abilities. The music therapist elucidated the infant's ability to express itself and interact. Together with the parents, the music therapist reflected on how their infant interacted in the moment. This heightened comprehensibility contributed to parents' understanding and coping mechanisms during the hospital stay, as they observed their infant actively engaging with music. Notably, without the therapist interacting with the infant, some parents may not have spontaneously considered singing as a means of interaction.

***826M:*** *But then, I thought above all that what was cool was when you [music therapist] came, and I could see that she responded… I didn't think she was responding to me, but then, when I began to be able to see it, it became almost even more important. Like, “oh, she understands this”… and choosing songs with care and all, that I want this […] I thought it was cool, especially when I noticed that she understood more than I thought about what it meant*.

***909M:*** *At the hospital, you [music therapist] demonstrated the signs he [baby] displayed in response to the song, and when you showed that, I understood that he was taking it in as well. But I probably wouldn't have spontaneously thought of singing*.

Numerous parents experienced a sense of wellbeing in their infants during singing and music therapy sessions. They described how their infants seemed to recognize their voices and presence, exhibiting relaxation when they engaged in singing. These encounters enhanced the comprehensibility for parents, affirming the significance of their singing in positively influencing their infants.

***969*****+*983F****: It was when we [parents] achieved that vibration in the chest bone that they really relaxed after a while*.

***134*****+*505F:*** *I experienced that the children felt secure and recognized the songs I sang*.

***930M**: I think she became a bit calmer […] this closeness made her more engaged, a shared experience maybe in some way*.

Some parents perceived singing as an intuitive way to communicate with the infant. They emphasized that when they sang, the infant heard and responded, establishing a connection similar to talking. Singing was seen as a natural and effective means of communication, allowing the parent to connect with the infant and observe their engagement through looking and listening.

***742*****+*115F:*** *When you sing, they [babies] hear a lot, you often get in touch like that when you sing. You don't sing into the air, you sing to them. In the same way as when talking*.

***551M:*** *I find it natural that one wants to sing; I think it becomes another way to communicate. You establish a connection with him [baby] and see that he's looking and listening*.

Several parents felt that their voices and presence meant something to their infant, something unique that no one else could provide for their infant that created a special bond between them and promoted their relationship. In this way their singing to their infant became highly comprehensible in their process of becoming parents.

***551F:*** *Just to create a bond, it will be something personal and it will be something he [baby] will recognize…he knows my voice, and knows what I'm singing… that's what you're creating here, a relationship […] There must be a difference between us and others. We are his parents*.

In interviews, numerous parents expressed that their engagements with the music therapist yielded new insights and a fortifying experience. They acquired a heightened comprehensibility regarding the positive effects of singing, acknowledging a substantial shift in perception and recognizing it as genuinely beneficial for their child.

***111M:*** *Yes! God yes, yes! I had some idea before and a feeling but then, when we met and I've been doing this research and this so…and so this book that you [music therapist] gave me. It has changed me completely, much more! So it's something that's really, really is good! Before I had a faint idea, but it was something I couldn't really put into words…but it felt good, that's how I felt! So it has definitely changed!*

A handful of parents not only shared their infants' experiences but also discussed their own emotions while singing and during music therapy sessions. Certain parents described this as a healing experience and a catharsis, the process of releasing strong or repressed emotions, thereby providing relief.

***477M:*** *I remember the second time we met, you [music therapist] sang “Trollmor” […] I just cried so much… Then, I recalled the entire childbirth […] Yes, it was like catharsis, like cleansing… I always become emotional with “Trollmor” […] When I think of that song, when I hear that song, it reminds me of the childbirth or the moment when you sang for us […] I just opened my heart and looked at what lies there*.

***348M:*** *I felt it, especially when we sang there in the emergency room… yeah, there were a lot of emotions that came out when we sang! Especially with these slightly sad lullabies, it was very liberating… because that's how you [parent] felt at that moment, you were kind of in grief, so it was really nice to sing some sad lullabies. Yes, I actually liked it! […] because there was maybe a lot that you [parent] couldn't put into words or say and so on… there were so many emotions that it was difficult to handle everything, I think, because it was like a storm inside!… then, to sing, it was kind of like relieving that pressure somewhere, actually!*

Numerous parents disclosed that intense emotions were evoked during music therapy sessions, whether through singing together during sessions, listening to the music therapist sing, or singing alone with their infants, contributing to a heightened sense of wellbeing. One parent spoke about the music therapy sessions as helpful in handling their grieving process.

***111M:*** *I used to feel good after our meetings, I became calm! It provided positive energy, so I appreciated that too! I used to be in a bad mood or sad or something when you [music therapist] came, I remember, but then… I felt better and calmer when you left*.

***551F:*** *It was quite emotional sometimes, it was. Especially with the loss of X. there… it was… you [parent] got some outlet for the grief, I think, through the music and that… it was a bit that it helped to move on from everything that happened. […] when you [music therapist] were about to come with your music sessions and enter our room, then there were mixed feelings like “no, what's going to happen now, am I going to collapse there?” but then when it was over, it was nice. You [parent] often had a positive feeling afterward… there was always a positive feeling afterward. In the end*.

#### 3.2.2 Control group

Also parents in the control group who sang felt that their singing had an impact on their infant in a positive way that made it comprehensible.

***927M:*** *She [baby] became calmer and felt safer, and she recognized what I sang to her in the womb. It felt like she became happier. When you sing, the child feels happier, safer and calmer! It's the same with adults*.

For certain parents in the control group, maintaining a regular singing routine for their infant in the NICU posed challenges, particularly in the early stages when the infant was fragile. Singing became more ingrained as the child reached 3–4 months of age and the stress associated with the hospitalization lessened. Parents sang both to soothe the child and, in retrospect, for their own comfort. Singing facilitated a sense of connection, involving physical touch and humming. However, initially, parents grappled with singing certain songs due to intense emotions, including worry, sadness, and fear.

***034M:*** *Yes [I sang] a few times but it was nothing regular…I also think it has to do with the fact that he [baby] felt so fragile in the beginning…it didn't feel natural to sing to him. […] it was rather when he was three to four months old, that was probably when I started singing. […] when all that had to do with the hospital disappeared, it was probably then that I could feel that I was here with my child*.

***338M:*** *It was actually meant to calm her [baby] down, but I think in retrospect it was mostly for myself. Because what you could do was to hold hands or something and sing and hum. […] I sang almost right from the start, but then I mostly did it when there was no one else around at first. I thought it was very difficult. I almost couldn't hum something like “Vem kan segla” without starting to cry, there were several songs that I couldn't sing without my voice starting to tremble, I had to choose songs that I could sing…. it was a lot that happened then! Much worry and sadness and fear and such!*

The initial hesitancy and challenges experienced by some control parents in singing to their infants evolved over time. As certain parents persisted in this practice, they began to notice positive changes, with infants displaying increased calmness and improved ease in falling asleep. These observations suggest a growing comprehension of the soothing impact of singing on the infants.

***123*****+*724F:*** *Initially, it felt more challenging… I didn't know if they [babies] noticed anything […] I eventually started noticing that they might become calmer at times and also fell asleep more easily*.

The hospital environment, characterized by decisions made by others, led certain parents to perceive a lack of authenticity in their parental role. Singing emerged as a distinctive and personal avenue for them to participate in the care, representing a significant opportunity to contribute actively to their child's wellbeing. This engagement with singing brought a positive and empowering dimension to their parental role, enhancing their sense of comprehensibility and contribution in the caregiving process.

***776M:*** *What I can also feel, when you were in hospital, you [parent] don't really get a chance to be a parent, someone else decides everything you have to do, but singing is something that only I can do and give this care instead of nurturing with medicines or with equipment in this way. You feel pretty bad as a parent in such a way that you don't know how to take care of your child. So it was nice when you could take care of your child in that way*.

To sum up, data collected from the intervention group indicate that singing, especially slightly sad lullabies like “Trollmor,” became a means of expressing difficult emotions, providing relief, and releasing internal pressure during a turbulent period. In this way, parental singing contributed to the comprehensibility of emotions related to childbirth and challenging moments, often associated with traumatic experiences. In contrast, the parents of the control group did not express the singing as catharsis and letting feelings go; instead, certain control parents expressed that when the singing evoked strong feelings, they hesitated to sing those songs to avoid eliciting such strong feelings. The results from the intervention group indicate that the parent's perception of their infant changed after meeting the music therapist. Initially, they did not fully perceive the infant as a responsive baby beyond basic needs. Singing played a transformative role, making the infant appear more human, establishing a stronger connection, and creating intimacy. Over time, as the infant grew, singing became a natural and meaningful way for the parent to assert their presence, strengthen the connection, and offer comfort, for both intervention and control groups.

### 3.3 Meaningfulness

#### 3.3.1 Intervention group

Many parents found singing for their infant a meaningful, comforting, and bonding experience, awakening deeper emotional connections. Despite the infant's minimal reaction, it created a connection and enhanced togetherness. Described as therapeutic, calming, and special, these moments' significance became more apparent in retrospect, emphasizing the depth of the bonding situation.

***020*****+*509F:*** …*it's very cozy to just sit there… that communication we [parents] got somehow, even if they [babies] didn't react a lot when we sang. They hear you […] that thing about forming a connection, so they can recognize your voice*.

***826M:*** *When she [baby] lay in the incubator and we sang, it was probably half or 75 percent therapy for me, just singing “Byssan lull” like some kind of mantra […] I felt that I became calm from it. […] it also served an important function for me, […] these moments became very special… we sat there in that chair and sang and sang. […] It becomes some kind of bonding situation in some way that I probably didn't understand then, but that I can see now*.

Numerous parents reported that when they sang, their infant became physically close, creating a sense of emotional closeness. They described this experience as deeply meaningful.

***930M: *** …*it was still like this… happiness to have this contact, it was more intense, it felt like she [baby] became more present, not just that she was close […] singing made it even closer*.

***808F:*** *It was cozier to be just me and X, we got a different connection somehow […] I felt it was very intimate*.

One parent also described the music therapy sessions as very relaxing moments and underlined particularly the harmonizing, uniting and blending effect of the monochord for them and how it strengthened their process of becoming a family.

***748M:*** *Yes, I thought the monochord was really cozy… it was very harmonious. It felt like we ended up in our own family bubble, that we became a unit, suddenly becoming a family*.

The CMT was acknowledged as emotionally supportive and empowering in the transition to parenthood. It offered comfort and tranquility, providing reassurance through the music therapist's guidance and dedicated time. Additionally, the CMT sessions contributed to the overall journey of parenthood, aiding in navigating challenges in the NICU and fostering a meaningful connection with the child.

***930M:*** *It was a comfort… it became like a calm. And then getting some tips on how I can use my voice and a little more to make it easier for both of us, that was reassuring. It was very nice because someone [music therapist] came and really took the time. […] And it gave a lot for oneself too, I think I became a bit strengthened, I didn't need to worry so much […] After each session, you [parent] felt that personally, you took another step… dared to believe in yourself a bit more in the new role as a mom. […] It has given so much… on the whole journey and to the person I has become as a mom today*.

***551M:*** *After all, we had to learn to be parents in the NICU. But it was difficult to get into that role because of all this stuff around. And then the song and this became a way to get close to your child, to get to know him, I think*.

The CMT sessions were described as recurring and anticipated moments, providing a broader meaning and context to the otherwise routine-driven existence at the ward. Certain parents described how music therapy sessions made their days in the hospital more meaningful.

***909M:*** *Then it [music therapy] became something to look forward to and to hang on to the thought of, getting to meet you [music therapist]. To go from my life before to sitting in that family room, it still became a different rhythm and to meet another person [music therapist] and to talk about something else that wasn't directly about pumping milk*.

***826M:*** *That it [music therapy] was also something that made the days go by and think about something other than ultrasounds and hearts and all that…but now we're going to have some music too*.

The connection between parents and music therapist during music therapy was regarded as meaningful by some parents. They highlighted the therapeutic nature of their interactions with the music therapist, emphasizing the value of dialogue to reflect on and organize their thoughts and feelings, thus contributing to the overall sense of meaningfulness in their relationship with their child. One mother especially emphasized it as important that the music therapist was not someone who was responsible for her infant's daily care and because of that the dialogue became more open and supportive in another way.

***057F:*** *I think you [music therapist] are very good; we have had very good conversations on all sorts of topics. So, the whole thing that in an unforced way you [parent] have the opportunity to reflect, feel, put into words how you think and feel, I think that's actually great!*

***909M:*** *And all that support I got from having a conversation with you [music therapist]! Who is not a healthcare professional, who does not look after X. in that way, but who has a different angle and something that we [parents] could choose ourselves and that I became very interested in*.

Music therapy and singing served as an emotional space where some parents could find respite from worries and other negative emotions and also respite from challenging and difficult thoughts about their situation. This emotional engagement was seen as contributing to the meaningfulness of their experience during the intervention.

***748F: *** …* very calming, especially this one with the monochord […] it was a chance to escape from reality and I thought that was very nice. […] I think you [parent] need it and it was also a nice break in everyday life, which is otherwise very routine driven in the NICU. So then you [parent] had something else to think about or talk about*.

***057F:*** *I thought it was cozy when you [music therapist] were there. It was nice, that little harp you had, it probably provides a calmness that is pleasant to be in. I appreciate it; it's somehow meditative, so I think it's great. It's the presence, it's something special about live music*.

Certain parents highlighted experiences of loneliness during their NICU stay. They emphasized that the dialogue and the chance to be present in the moment, free from demands, during CMT sessions, were profoundly supportive and meaningful for them. This finding underscores the role of CMT in addressing emotional challenges and fostering a meaningful connection for parents in the NICU.

***348M:*** *I thought it was great, really great! Partly it was nice to sing and get inspiration for it, but then I got so much support in our conversations! It was so incredible; it really meant a lot because… I was also quite lonely there in the ward*…*so it was great when you [music therapist] came and we actually got to talk a bit. So that it wasn't just the song, but also that you [parent] got a moment to sit and reflect on everything and talk a little, you actually needed that*.

It seemed that music therapy, the contact with the music therapist and starting to sing to their infant gave several parents a context that made their stay in the NICU more meaningful. Music therapy in the NICU was not something that they had expected but that became very important to them during hospitalization with their infant. They described the calming effect and the closeness as highly meaningful.

***348M:*** *It's not something you [parent] might come up with yourself, that it's SO good to sit down and sing with your child. […] so as to get inspiration to sit down and sing for a while. I thought it was really, really nice!…and just that you [parent] took that moment with your child, it actually became such a small quality moment. […] because it wasn't just the music, but the fact that you sit down close to your child and you get in touch and you notice that the child relaxes and maybe falls asleep, so it's really nice!*

***969*****+*983M:*** *I think it's good too, I would not have expected that in the NICU… I really believe that the calming effect it has on both parents and children is very important in the situation you [parent] find yourself in!*

After discharge from the NICU all parents reported that they continued singing to their infant at home. Singing had seamlessly become an integrated part of their family life, and they expressed profound appreciation for the meaningfulness of this practice. This finding suggests that the practice of singing persisted as a meaningful and valued aspect of their family routine post-discharge.

***111F: *** …*now she [baby] grows up with singing all the time…and our voices and with the same song and that, it calms down at once. She becomes happier*.

***974M:*** *Now I think it [singing] comes so spontaneously, now it comes all the time. I don't think you think about it, but you sing quite a lot [to father]. Me too! I sing her name and when changing diapers. I think you [to father] sing to her more than you talk to her*.

***508M:*** *He [baby] reacts, he smiles and laughs, he moves. […] I respond to his sounds, imitate him and sing to his sounds or make up songs based on what he says*.

***969*****+*983M:*** *I sang a lot for her [baby] last week when she was going to have surgery. I sang when they were taking a blood sample and when she needed to be still for an examination. It was clear that she calmed down when I sat there and sang. But I also sing sometimes and play around with them [babies], and then they smile and laugh occasionally*.

One parent told about that the continued singing at home awakened memories of songs from her own childhood and it became meaningful to share them with her infant.

***551M:*** *And then other songs come up that you yourself [parent] have learned when you were little, that you sing…you are drawn back to your childhood. So I think it's wonderful and very nice to be able to sing my favorite songs*.

A number of parents characterized singing at home as a means to support and nurture the evolving relationship with their child, highlighting its role in strengthening their parental identity. This finding underscores the meaningfulness of singing beyond the hospital setting, positioning it as a purposeful and identity-affirming practice for parents.

***808M:*** *To give security…belonging… it becomes a cozy moment, and you connect with each other and it becomes intimate. […] this [singing] is something that you will really continue with, I feel, it is a very natural part […] yes I think that it furthers our relationship*.

***551F:*** *You have your songs [to mother] and I have my songs…you [parent] get a different connection, your personal connection to X through what you sing. […] Yes, but somewhere it [singing] has become his…a part of him thanks to this thing we've been doing, the singing. It becomes a bit like a part of him. So, you [parent] like to keep doing it, you don't want to skip it*.

In discussing singing at home, several parents emphasized the multitude of emotions it evoked, both for their infants and themselves. These findings underscore the meaningfulness of singing in the home environment, illustrating its capacity to elicit a rich emotional experience for both parents and their infants.

***236F:*** *Yes, I become happier myself! It's somehow enriching to spend time with X in several different ways. […] it's happened that I've had the feeling that he sings along, tries to sing along and also there are certain songs that he can laugh at and stuff like that*.

***930M:*** *She [baby] becomes happy, I become very happy. […] now you get a completely different connection and then there becomes a completely different closeness […] we also talk a lot during the day, but the singing in particular is a little more…yes…a little more joy…that is, feelings*.

Furthermore, the practice of singing at home evolved into a shared activity for some parents, adding a layer of meaningfulness and enjoyment for both parties. This finding highlights the collaborative and enjoyable nature of singing within the home environment.

***020*****+*509M:*** *It's also so fun that it [singing] has become something that me and X [dad] do together[…] it's positive for us to sing as well, it's fun to sing*.

The majority of parents regarded music therapy, especially singing, as an integral supplement to the medical care received in the NICU. In hindsight, they viewed singing as a meaningful addition to the routine care provided in neonatal wards, acknowledging its potential to offer parents a break from the customary tasks of caregiving and medical assessments. This finding underscores the perceived significance and positive contribution of singing to the overall neonatal care experience.

***930F:*** *I definitely think it [music therapy] can be a good adjunct to “usual care”. I also think that it can be a welcome feature for the parents as it can be a break from the usual routines of care and medical checks*.

***134*****+*505F:*** *I thought the contact with you [the music therapist] was good. We probably wouldn't have thought about the meaning of singing without this contact, so it was appreciated. Then we could also talk about other things too, which was good. I absolutely think that this [music therapy] is a good feature of the medical care in neonatal-wards*.

A handful of parents regarded singing in neonatal care as an essential component of the healing process, wanting it to be available to all families to support parenting and foster coherence. They perceived singing as a valuable tool for establishing a close connection between parents and their infant, providing a unique and positive means of interaction. Stressing the importance of comprehending and promoting the significance of singing in a pedagogical manner, parents believed it could enhance therapeutic benefits for both parents and infants. This finding highlights the meaningful role parents attribute to singing in the context of neonatal care, emphasizing its potential positive impact on the parent-child relationship and the overall wellbeing of families.

***862M:*** *I think this [singing] is a very important form of a part of the healing process that is very important! […] So, I hope this [music therapy] gets into neonatal care and like really, really all babies even full term would need it. Because I think singing can be a tool to get close to your child! […] And that's why I think, or I wish, that you could work more with singing as a healing method… Yes, as a tool for parenting… it is. You can hum and be tone deaf but still it's nice because you have a moment with your child…so…I think it's something you should have more of!*

***862F:*** *[…] it is, and if you can encourage and help to encourage others in a pedagogical way so that you understand how important it [singing] can be and how good it can be…and help on the trot, so to speak, with that bit, I think it's good! Really!*

#### 3.3.2 Control group

Certain parents in the control group who sang described it as meaningful and important in their journey of parenthood.

***776M:*** *I felt like a good parent. I think it's something that, as a parent, I have to sing for my children and when you do it, it just feels good, you feel good and it's very cozy!*

Several control group parents, upon returning home from the hospital, initiated singing with their infants, with some adopting this practice from the outset. Upon reflecting on their motivations, these parents highlighted the significance of shared emotions, viewing singing as a communicative tool and acknowledging its importance for the child's development. This discovery underscores the meaningfulness that parents attribute to singing, emphasizing its role not only as a form of communication but also as a deliberate and purposeful activity intended to nurture a connection with their infants and support their developmental journey.

***524M:*** *It [singing] is like a language and a communication between us. […] Yes we are on the same emotional level, we share feelings through it. When I see that he [baby] is sad, I hug him and sing a sad song…when we are happy, we sing something happy. […] I think it affects in different ways, his well-being too…he feels safer and it's also a fun thing we do together*.

***034M:*** *That it's fun, I notice that he [baby] gets excited when I sing…he thinks it's great fun. […] I think that the more you stimulate your children with a song, talk or something, I think it's clear that you stimulate the brain, they [babies] get impressions, and they feel a sense of belonging… I think that's super positive!*

Control parents in the study gathered inspiration from various sources, such as their professions, literature, and interactions with healthcare professionals. This multifaceted approach to finding inspiration highlighted the meaningfulness parents associated with incorporating a variety of influences into their singing practices. Also, the weekly contact with the music therapist gathering the diaries and then talking about everyday challenges fostered a therapeutic relationship and for some control parents were also supportive to them beginning to sing.

***927M:*** *Maybe from you! But I did it [singing] with my first child too, it's normal, I think. What you did with the first child, you also do with the second. […] I am also a nanny and have knowledge from my profession. I do it in my job all the time and with big brother. And now I do it all the time singing to her. Then I've read a lot of books about how the child reacts and how the child becomes if you sing and if you talk to them and how they develop and so on. It is natural*.

***338M:*** *Because it felt like it [singing] was the only thing I could do for her [baby]. I hardly dared to touch her…and it was hard to touch her, there was so much stuff and it felt like I couldn't do anything, so I took the ones [songs] I could, there was a lot of humming rather than singing. But then I contacted a colleague who holds Baby Rhyme sessions and asked for some songs. […] At H [other hospital] I thought they were encouraging and a doctor there was humming to herself when she removed the tape to calm down and it was a bit inspiring and then it felt like then I can also do this if even she [the doctor] does it! […] they [nursing staff] encouraged us to talk or sing…and since we couldn't do much, they thought it was important for us to sit there and talk or sing. I found it difficult to sit and talk so I preferred humming*.

The collective engagement in singing proved to be a meaningful activity for one control group family, fostering a shared experience between both parents.

***123*****+*724M:*** *But it was still very nice that we're doing something [singing] together, that I'm doing something for the children, it felt very cozy. […] We sat in our own armchairs, each with a child on our stomachs, and sang together*.

Several parents in the control group acknowledged the value of seeking guidance and support from a music therapist or engaging in music therapy during their NICU stay, emphasizing the meaningfulness of these interactions.

***034M:*** *I think it's great, for some music is not natural at all, to sing to your child […] I think it's good both to get those who already have it in them started but maybe also to inspire those who music doesn't come naturally at all. […] I probably would have needed that nudge because I thought I ought to sing for him [baby]*.

***927M:*** *It's good that it [music therapy] exists! You [parent] get support so that you feel safer if you think it is difficult to sing. You might feel more secure and that you are doing something that is good and that even if you might be doing it yourself, it might be easier if someone else [music therapist] helps*.

Concerning the last key concept of SOC, meaningfulness, numerous parents from the CMT-intervention group expressed how music therapy during their time in the NICU gave them something to hold on to. CMT became a source of emotional-social support and strength in the journey of becoming a parent, offering valued personalized accompaniment, comfort, calm, reassurance, advice, and dedicated time. Moreover, CMT sessions played a meaningful role in the overall experience of parenthood, helping parents navigate challenges in the NICU and fostering a deep connection with their infant. These sessions served as a precious break from the stress of the NICU, contributing to a sense of meaningfulness and benefiting both parents and the infant. In the control group, parents also talked about singing as a meaningful way to connect with their infants. They talked about how other sources inspired them to sing and that the weekly dialogue with the music therapist collecting diaries was supportive. The control parents, when asked, also reflected upon which support they would have needed from the music therapist if provided.

Even though we didn't specifically ask about negative experiences, the results do not reflect any negative or critical quotes from the parents in either the intervention or control group.

## 4 Discussion

The aim was to investigate the potential impact of CMT in the NICU on enhancing the sense of coherence in parents as they sing to their preterm-born infants during kangaroo care. Additionally, we explored whether CMT had any significance on family life after the NICU stay. Our findings indicate a positive influence of CMT on intervention parents' experiences of singing to their preterm infant during kangaroo care and by increasing their sense of coherence in terms of manageability, comprehensibility, and meaningfulness. All parents in the intervention group experienced that CMT was a music therapy method that strengthened them as parents and was helpful in supporting the incipient relationship building with their infant when engaging in singing. The inductive analysis revealed recurring patterns within the dataset, identifying key thematic elements associated with positive emotions and experiences. These identified themes encompassed *feelings of safety, closeness, empowerment in parenting*, and *healing*, merging into the overarching category and framework of the SOC so that after the first inductive stage of analysis, a deductive confirmatory stage was embarked upon in which we tested the categories found according to the existing framework of SOC.

Similar experiences of CMT have been documented elsewhere (Kehl et al., 2021; Haslbeck, 2014; Haslbeck et al., 2021). Kehl et al. (2021) reported relaxation for both infants and parents during singing as a key finding, along with parents associating relaxation with the intimacy it fostered between them and their infant. Our study corroborates these findings by demonstrating parental singing as a practical and effective tool to soothe the infant, especially in the NICU. The results indicate that singing may serve, not only to calm the baby but also the parents, as a valuable strategy for them to manage their stress and create a calming atmosphere for both them and the infant.

In discussing the importance of safety for relaxation and the subsequent creation of closeness as a foundation for interaction, it is essential to draw upon the insights provided by Porges (2011) and Sanders and Hall (2018). According to their research, when parents feel secure, they are better equipped to relax with their infant, fostering a sense of closeness through activities such as singing, engaging in small talk, and gentle caressing (interaction). This interaction initiates a cycle of co-regulation, where both parent and infant contribute to each other's relaxation, resulting in an immediate reduction of stress, and a broader sense of meaningfulness derived from the shared experience of closeness. In our study, participants often described experiencing what Stern (2004) terms “now-moments” during relaxed and intimate interactions, where parent and infant emotionally attuned to one another, thereby strengthening their relationship. These “neonatal moments of meeting” are foundational for bonding after birth (Bruschweiler-Stern, 2009) and represent meaningful therapeutic moments of change in CMT. Within these moments, parent and infant synchronize and attune through communicative musicality (Malloch and Trevarthen, 2009), aligning in pulse, timing, and vitality forms (Haslbeck, 2014). We interpret the SOC as reflecting the closeness and relationship between infant and parents, paralleling the synchronized interactions that foster these valuable “now-moments.

In our study, intervention parents describe such moments with words like closeness, presence, bonding, and intimacy when feeling emotionally and physically close to their infant. Similar findings were reported in Haslbeck et al. (2021), where parental quotes echoed these sentiments. Despite the passage of several years since their interviews, parents vividly recall how CMT helped them and their infants relax, fostering empowerment and strengthening the parent-infant bond.

Parents in the intervention group emphasized the importance of their relationship with the music therapist, particularly highlighting responsiveness as a crucial aspect of CMT. This aligns with the findings from a previous study (Haslbeck, 2014), which discussed the positive impact of inter-subjectivity in music on relaxation, empowerment, and interaction for both parents and infants. Indeed, CMT, being supportive in creating a space for undemanding responsive inter-subjectivity, is viewed positively from a parental perspective. Additionally, our results support the need for psychosocial support for parents in early intervention programs, as emphasized by a meta-analysis deeming mere education insufficient (Benzies et al., 2013). Most intervention parents found it helpful to initiate and sustain singing with their infants. Some parents acknowledged the value of the music therapist's expertise in facilitating meaningful connections through music, highlighting that they might not have spontaneously thought of singing without the music therapist's guidance. Supportive elements varied among parents, including singing with the music therapist, understanding the importance of singing, receiving emotional support during shared moments, and the opportunity to experience their infant alongside the music therapist. The significance of the music therapist's undemanding approach is also emphasized, aligning with Shoemark (2017), who underscores the music therapist's role in supporting parents during NICU admission.

Additionally, intervention parents in our study emphasize the music therapy and the relationship with the music therapist as emotionally supportive. Emotional care, crucial in the NICU, emphasizes the therapeutic relationship for sharing presence and compassion (Bruschweiler Stern, 1998). Coughlin (2021) emphasizes Compassionate Family Collaborative Care, promoting emotional support for infants and parents through collaboration between professionals and parents. This approach resonates with parents' experiences of CMT in our study, suggesting that CMT offers vital emotional support, including comfort, calm, reassurance, and dedicated time for parents. One mother described how a particular song (“Trollmor”) during music therapy sessions evoked deep emotional responses, serving as a cathartic expression of childbirth and challenging moments. Another parent found singing together with the music therapist liberating, especially when singing slightly sad lullabies. It became a way to articulate difficult emotions, providing relief. Yet another parent mentioned that singing during CMT sessions helped them manage sorrow. Similar emotional release through singing is reported by Palazzi et al. (2020), where a mother describes singing to her infant as an emotional release that made her feel better, and the music therapy intervention helped establish a more intimate connection between her and her infant. McLean (2016) emphasizes the emotional support provided by the music therapist in facilitating the parents' ability to engage with their infant through singing and music. Epstein and colleagues in their interview study (2023), describe how parents experience music therapy as an emotional haven.

In our study, several fathers found singing to their infant to be a significant bonding activity during the hospital stay and after discharge, which held deep meaning for them. Consistent with our findings, parents in the aforementioned studies also emphasized singing as a proactive contribution to their infant's wellbeing during the hospital stay. Singing was seen as something they could do for their infant (McLean, 2016; Palazzi et al., 2020).

In the control group, parents who sang generally described positive experiences and found it meaningful while interacting with their infant despite not receiving the same level of support from the music therapist as the intervention group. They felt a sense of cohesion within the family through parental singing, finding joy in it even without closer musical guidance. All parents in the intervention group continued to sing at home. However, perhaps motivated by the study's recruitment information, this also applied to several parents in the control group. In this way, singing served as a bridge from the NICU to home life, strengthening family bonds. This aligns with the findings of Haslbeck et al. (2021), where parents reported that singing remained significant in family life many years later. This suggests that a CMT resource-oriented approach fosters emotionally nurturing interactions, empowering parents and strengthening family bonds over the long term. It also aligns with two other qualitative music therapy studies where parents report that they continue to use singing to their infant at home as a resource and parenting agency (Epstein et al., 2023), e.g., to soothe and facilitate self-regulation in their infant (Ghetti et al., 2021).

However, the most notable contrast between parents in the intervention and control groups was the lack of emotional support experienced by most control group parents. Since the variability in responses is much more constrained in the control group compared to the intervention group, generalizing the findings is challenging. However, if the material is representative, this disparity aligns with Winnicott's concept of a holding environment (Winnicott, 1971), which is essential for patient healing (Sanders and Hall, 2018). Creating such an environment is crucial in the NICU for fragile parents and infants (Stern, 1998). The emotional care provided by the music therapist facilitated healing and catharsis for intervention parents, whereas control parents lacked this support. Some intervention parents attributed the introduction of singing as a supportive tool, in their new role as parents, to the therapist, highlighting the latter's role in fostering meaningful connections through music.

Parents in both the intervention and control groups stressed the importance of integrating music therapy into neonatal care, citing its significance in supporting both infant development and parental transition into parenthood, consistent with findings by Haslbeck et al. (2021). Some participants suggested extending music therapy not only to NICU families but to all new parents, particularly emphasizing the value of singing to their infants during early parenthood. One mother even described singing to her infant as a healing intervention. Indeed, prenatal singing is associated with improved maternal mood, wellbeing, and mother-infant bonding (Wulff et al., 2021), as well as postnatal benefits such as reduced maternal stress, fewer neonatal crying episodes and less infantile colic, even in cases of prematurity (Persico et al., 2017).

In the Time Together program, Shoemark (2018) has devised a strength-based approach aimed at encouraging NICU parents to sing to their preterm infants after just one session with a music therapist. The findings from the control group in our study suggest that this approach may suffice for parents with a musical background who are already singing. However, some parents in the control group expressed that it would have been helpful to receive assistance in initiating singing and to have someone to share the experience with.

CMT sessions with the intervention group played a meaningful role in the process of becoming a parent, addressing NICU challenges and deepening the parent-child connection. This aligns with McLean et al. (2018), and with Epstein et al. (2023), regarding on parents' experiences of music therapy in the NICU, highlighting infants' responses in musical interaction as validating and strengthening in the process of becoming a parent. In our study, some parents initially did not fully perceive their child as responsive, but singing played a transformative role, the infant appeared more human, it created intimacy and established a stronger connection. Experiences like these are significant in the NICU, offering parents a sense of comprehensibility and empowerment (Flacking et al., 2012). In this way our study aligns with Ettenberger et al. (2016), where parents refer to music therapy experiences as giving insights into how to relax and stimulate their infant through music, giving them a feeling of empowerment. Additionally, several intervention parents in our study highlighted the significance of participating in dialogues during music therapy sessions as supportive when recognizing their transition into parenthood.

Our results indicate that parents in the intervention group expressed how their interaction with the music therapist during music therapy sessions heightened their awareness of their infant's communicative skills. Furthermore, the intensity of experienced beneficial effects for the parents varied between the intervention and control groups. These findings are consistent with previous results from the Singing Kangaroo trial (Partanen et al., 2022). They emphasize the enhanced caregiver sensitivity resulting from the interactive qualities of CMT and suggest that this may be the driving component behind the enhancement of auditory processing in the brains of CMT infants compared to control infants. Rather than solely focusing on the amount of parental singing, they argue for a broader understanding of the effects of CMT, which includes improved parental self-esteem and sensitized caregiving. This is in line with the findings of Doiron et al. (2021) and Loi et al. (2017), who highlight the importance of enhanced parental self-esteem and sensitized caregiving for the wellbeing of both infants and parents, and their association with improved long-term infant development. Our results may indicate improving supporting the relationship between parents and infants during hospital care in the NICU. Although we didn't specifically ask about any negative influence on the relationship between infant and parents due to singing, it seems that the components in CMT are well-suited to embrace closeness for the whole family. Additionally these experiences of closeness through singing develop and reach out to the family's daily life when coming home after discharge. Therefore, we argue that CMT appears to be a relevant intervention in the NICU for enhancing self-esteem, fostering empowerment, and supporting in their transition to parenthood, which, in turn may contribute to the development of the premature infants. Despite its limitations, the methodological rigor and innovative approach contribute to a foundational understanding of parental experiences, which may inform future research and practice in neonatal care. Future research in this field could make an even deeper investigation about which are the core components in a child and family developmentally supportive music therapy such as CMT when it comes to supporting closeness and wellbeing for the whole family. Another important aspect to investigate is how the collaboration between health professionals and music therapists can support this. Future longitudinal research on parental and infant wellbeing is warranted, assessing SOC, bonding, attachment, parental skills, and early interaction with appropriate, sensitive quantitative outcome measurements, as well as qualitative studies. Furthermore, more research is needed on follow-up music therapy after discharge to explore how CMT can support both the family and the infant's long-term development.

## 5 Conclusion

Although parents in the control group experienced joy and a certain degree of sense of coherence (SOC) when singing together with their infant, it was primarily the parents in the intervention group, who had participated in Creative Music Therapy (CMT), who experienced a strong sense of coherence SOC across all three of its categories: manageability, comprehensibility, and meaningfulness.

In conclusion, the SOC framework, with its components of manageability, comprehensibility, and meaningfulness, reflects the dynamic relationship between infants, parents, and their shared closeness. This connection resembles the synchronized interactions that occur during intimate moments, creating meaningful “now-moments” that strengthen their bond. This synchronicity, particularly in the stressful NICU context, may facilitate healing within the vulnerable family unit. This study explores how parental singing influences SOC in the NICU and beyond, offering insights for optimizing family-centered care for prematurely born infants. Even though the data is not sufficiently representative to draw general conclusions about how parental singing influences SOC in the NICU and beyond, it provides valuable insights into the potential benefits of integrating parental singing into family-centered care for prematurely born infants. To build on these findings, future studies should focus on examining the long-term effects of such interventions and evaluating their scalability. This could further enhance our understanding and inform the development of more comprehensive and effective care strategies in neonatal settings.

## Data Availability

The original contributions presented in the study are included in the article/Supplementary material, further inquiries can be directed to the corresponding authors.
