# Supplementary material for: Parental singing during kangaroo care: parents' experiences of singing to their preterm infant in the NICU
Source: Front Psychol. 2025 Feb 4;16:1440905. doi: 10.3389/fpsyg.2025.1440905 (PMC11832525; doi:10.3389/fpsyg.2025.1440905)
Supplement: Supplementary file 1 [file Data_Sheet_1.pdf]

Interview questions Singing kangaroo intervention group

How did you experience the contact and closeness with your baby when you sang to him/her/them during the time in the hospital?

Please describe the feelings you experienced when you sang to your baby in the hospital and how you felt the baby reacted to your singing.

Have you continued to sing to your baby after coming home? In what situations do you sing?

Please describe how you feel when you sing now. Can you see that your baby react to you singing? If so, in what way? Movements, gestures, facial expressions, vocalizations, sounds?

Do you have any idea whether singing to him/her/them can affect your child's development? If so, how? Relationship, attachment, emotional development, language, cognition, motor skills?

Has singing for your baby affected your relationship in any way? If YES in what way?

Within your participation in the study, you met a music therapist. How do you view the support/time/inspiration/information you received?

What were your feelings during music therapy sessions?

Have you learned anything new when it comes to singing/humming/vocalizing and small talking with your baby during the music therapy?

How do you see the possibility that music therapy could be a supplement to the medical care in neonatal wards?

Interview questions Singing kangaroo control group

Did you sing to your baby during the time in the hospital? If yes, what were your feelings during singing? If no, did you do anything else to connect emotionally with your baby?

If you sang, where did you get inspiration from?

Are you singing to your baby now at home? If yes, what are your feelings during singing and how do your baby react? Do you think that singing can effect your baby`s development in any way? The relationship between you? If yes, in what way?

How do you view the possibility to meet a music therapist on the neonatal ward?

How do you view the possibility that music therapy could be a complement to the medical care in neonatal wards?
